# Pulmonary disorder induced by cross‐linked polyacrylic acid

**DOI:** 10.1002/1348-9585.12369

**Published:** 2022-12-02

**Authors:** Yasuyuki Higashi, Yasuo Morimoto, Chinatsu Nishida, Taisuke Tomonaga, Hiroto Izumi, Ke‐Yong Wang, Hidenori Higashi, Ryohei Ono, Kazuki Sumiya, Kazuo Sakurai, Kei Yamasaki, Kazuhiro Yatera

**Affiliations:** ^1^ Department of Respiratory Medidatacine University of Occupational and Environmental Health Fukuoka Japan; ^2^ Department of Occupational Pneumology Institute of Industrial Ecological Sciences, University of Occupational and Environmental Health Fukuoka Japan; ^3^ Shared‐Use Research Center, School of Medicine University of Occupational and Environmental Health, Japan Fukuoka Japan; ^4^ Department of Environmental Health Engineering Institute of Industrial Ecological Sciences, University of Occupational and Environmental Health Fukuoka Japan; ^5^ Department of Chemistry and Biochemistry The University of Kitakyushu Fukuoka Japan

**Keywords:** cross‐linked polyacrylic acid, organic polymers, pulmonary toxicity

## Abstract

**Objectives:**

Organic polymers are materials widely used in our daily lives, such as daily necessities, foods, and medicines. There have been reports recently that cross‐linked polyacrylic acid (CL‐PAA) can possibly cause serious lung disease. We investigated whether intratracheal instillation of CL‐PAA causes pulmonary disorder in rats.

**Methods:**

Male F344 rats were administered low (0.2 mg/rat) and high (1.0 mg/rat) doses of CL‐PAA intratracheally and were dissected 3 days, 1 week, 1 month, 3 months, and 6 months after exposure to examine inflammatory and fibrotic responses in the lungs. Only the high‐dose specimens were subjected to ultrasonic dispersion treatment of the administered material.

**Results:**

There was a dose‐dependent increase in the total cell count, neutrophil count, neutrophil percentage, lactate dehydrogenase (LDH), surfactant protein D (SP‐D), cytokine‐induced neutrophil chemoattractant (CINC)‐1 and CINC‐2 values in bronchoalveolar lavage fluid (BALF) from 3 days to at least 3 months after intratracheal administration of CL‐PAA. Heme oxygenase‐1 (HO‐1) in lung tissue was also persistently elevated from 3 days to 6 months after exposure. Alkaline phosphatase (ALP) in BALF was elevated at 3 days and 1 month after exposure only in the high‐dose group. Histopathological findings in lung tissue showed inflammatory and fibrotic changes from 3 days after administration, and we observed obvious inflammatory changes for up to 3 months and fibrotic changes for up to 6 months.

**Conclusion:**

Intratracheal administration of CL‐PAA induced persistent neutrophilic inflammation and fibrosis in the rats' lungs, suggesting that CL‐PAA may have inflammogenic and fibrogenic effects.

## 1 INTRODUCTION

Cross‐linked polyacrylic acid (CL‐PAA) is a generic term for organic polymer compounds synthesized using acrylic acid as the monomer, and it is an organic material. The polymer's viscosity and water absorbency are increased when it is cross‐linked by a cross‐linking agent, and because of this difference in properties, it is used in various applications such as paints, shampoos, and other daily necessities, and in additives for food and pharmaceuticals.^1^ In April 2017, the Ministry of Health, Labor, and Welfare in Japan announced the occurrence of pulmonary diseases in workers handling materials composed mainly of CL‐PAA. Although the targeted workplaces were small, six people developed the disease, all of whom had been exposed to CL‐PAA, and many of them developed interstitial pneumonia with various lesions such as pulmonary emphysema, pneumothorax, fibrosis, and traction bronchiectasis in a short period of time around 2 years from the start of exposure. At present, however, we do not know whether CL‐PAA did cause pneumoconiosis.

Compared with inorganic materials such as asbestos and crystalline silica, organic materials are less likely to cause pneumoconiosis, including interstitial pneumonia, but in recent years, it has become known that hypersensitivity pneumonitis is caused by allergic reactions due to inhalation of organic materials. It is also known that continuous chronic exposure to antigens causes fibrosis;^2^ that is, secondary fibrosis via allergic reactions.

In addition to the Japanese case mentioned above, there was a case in South Korea where more than 100 people died of interstitial pneumonia after inhaling polyhexamethylene guanidine phosphate (PHMG‐p) due to the use of a humidifier infused with a disinfectant, indicating that the organic material directly caused interstitial pneumonia.^3^ Thus, some organic materials are thought to cause inflammation and fibrosis in the lungs by inhalation.

Therefore, we investigated the inflammatory and fibrotic potential of CL‐PAA in the lungs and its mechanism by observing a rat model in which CL‐PAA was administered intratracheally over time.

## 2 MATERIALS AND METHODS

### 2.1 Sample polymer

We used CL‐PAA (306223 poly [acrylic acid]): average Mv ~ 3 000 000 (Sigma‐Aldrich Co. LLC., St. Louis, MO, USA) and mixed the polymer with distilled water, slowly stirred for 40 min (Mag‐Mixer MF820 or MD300, Yamato Scientific co., Ltd., Tokyo, Japan). There was a concern that coarse agglomerated particles would obstruct the airway in the high‐dose group, so it was ultrasonically dispersed at 23 kHz for 10 min (ASU‐10D, Taiyo Company Co., Ltd., Osaka, Japan). The fundamental characteristics of CL‐PAA are summarized in Table 1. The polymer used in this study had a weight average molecular weight (M_W_) of 6.49 million as measured by multiangle light scattering coupled with field flow fractionation (FFF‐MALS) (Wyatt Technology Europe GmbH, Dernbach, Rheinland‐Pfalz, Germany). The secondary diameter of the polymers was 3.00 μm (median) and 2.31 μm (mode) in the testing suspensions were measured by a total holographic counting system using xSight (Spheryx, Inc., New York, NY, USA). The hydrodynamic diameter of the polymers was 1.62 μm measured by the dynamic light scattering (DLS, DynaPro NanoStar, Wyatt Technology Corp., Santa Barbara, CA, USA). The mass median aerodynamic diameter (MMAD) in Aerosol was 0.92 μm measured by Low‐Volume Air Sampler (Model AN‐200, Andersen Type, TOKYO DYLEC CORP., Tokyo, Japan). The mass concentration in aerosol was 1.76 mg/ml determined by the filtered weight of particles and the volume of air. The expected number concentration in aerosol condition was 3.01 × 10^6^ particles/ml measured by a particle counter (KC‐52, RION Co., Ltd., Tokyo, Japan). The volume concentration and the effective density of aerosol condition were calculated at 1.23 × 10^−3^ ml/m^3^ and 1.43 g/ml, respectively.

**TABLE 1:** Physiochemical characterization of the polymer used in this study

Name	Polyacrylic acid (PAA)		Monome	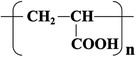
CAS number			9003‐01‐04	
Bulk	Purity		≦100% (Benzene 0.5%)	
	Molecular weight	Weight average molecular weight (Mw)	6 490 000	
		Viscosity average molecular weight (Mv)	3 000 000(average)	
	Cross‐linking		~0.1%	
	Appearance		Powder, white color	
Suspension	Secondary particle diameter		3.00 μm(median), 2.31 μm(mode)	
	Hydrodynamic diameter		1.62 μm(average)	
Aerosol	Mass median aerodynamic diameter		0.92 μm	
	Mass concentration		1.76 mg/m^3^	
	Number concentration		3.01 × 10^6^ particle/L	
	Volume concentration*		1.23 × 10^−3^ ml/m^3^	
	Effective density**		1.43 g/ml	

*Note*: *Volume concentration

Volume concentration_aerosol_

= Particle volume (ml/particle) × Number concentration (particle/m^3^)

= 4.08 × 10^−12^ × 3.01 × 10^9^

= 1.23 × 10^−3^ ml/m^3^.

**The effective density (g/ml) (aerosol)

ρ_Eff_aerosol_ = (Mass concentration (g/m^3^))/(Volume concentration (ml/m^3^))

= (1.76 × 10^−3^)/(1.23 × 10^−3^)

= 1.43 g/ml.

### 2.2 Animals

Male Fischer 344 rats (8 weeks old) that were used for exposure to polymers were purchased from Charles River Laboratories International, Inc., Kanagawa, Japan. The animals were housed at the Laboratory Animal Research Center of the University of Occupational and Environmental Health for 2 weeks with free access to commercial feed and water. All the procedures and animal handling were performed in accordance with the guidelines that are described in the Japanese Guide for the Care and Use of Laboratory Animals and were approved by the Animal Care and Use Committee, University of Occupational and Environmental Health, Japan (animal studies ethics clearance proposal number: AE18‐021).

### 2.3 Intratracheal instillation

Doses of 0.2 mg (0.8 mg/kg BW) and 1.0 mg (4.0 mg/kg BW) of CL‐PAA were dissolved in 0.4 ml of distilled water and injected intratracheally in rats (12 weeks old) only once. A control group received 0.4 ml of distilled water.

### 2.4 Animals following intratracheal instillation

There were five rats in the exposure group and the control group at each time point. Rats were sacrificed under anesthesia by inhalation of isoflurane (Pfizer Japan, Tokyo, Japan) at 3 days, 1 week, 1 month, 3 months, and 6 months after intratracheal instillation. Body and lung weights were measured at autopsy. The blood of the rats was removed from the abdominal aorta, and the lungs were perfused with normal saline. The right lung was repeatedly infused with normal saline under a pressure of 20 cmH_2_O, following fluid recovery two times, while the left main bronchus was clamped. Between 5 and 12 ml of the recovered fluid (bronchoalveolar lavage fluid; BALF) was collected by free fall into tubes, and then the right and left lungs were divided. The third lobe of the right lung after recovery of BALF was homogenized and used for Heme oxygenase‐1 (HO‐1) evaluation. The left lungs were inflated and fixed by 10% formaldehyde under a pressure of 25 cmH_2_O for use in histopathological evaluation.

### 2.5 Cytospin analysis of inflammatory cells, measurement of lactate dehydrogenase (LDH), and alkaline phosphatase (ALP) in BALF

The BALF was centrifuged at 400*g* at 4°C for 15 min, and the supernatant was transferred to a new tube to measure the LDH and cytokines. The pellets were washed by suspension with polymorphonuclear leukocyte (PMN) buffer (137.9 mM NaCl, 2.7 mM KCl, 8.2 mM Na_2_HPO_4_, 1.5 mM KH_2_PO_4_, and 5.6 mM C_6_H_12_O_6_) and centrifuged at 400*g* at 4°C for 15 min. After the removal of the supernatant, the pellets were resuspended with 1 mL of PMN buffer. The number of cells in the BALF was counted by ADAM‐MC™ (AR BROWN CO., LTD, Tokyo, Japan), it is an automated cell counting device, and the cells were splashed and fixed onto a glass slide using cytospin and stained with Diff‐Quik® (Sysmex CO., Kobe, Hyogo, Japan), then the number of neutrophils and alveolar macrophages were counted by microscopic observation. The released LDH activity in the BALF supernatant was measured by the Cytotoxicity Detection Kit^PLUS^ (LDH) (Roche Diagnostics GmbH, Mannheim, Nordrhein‐Westfalen, Germany) according to the manufacturer's instructions. LDH activity was estimated using a standard curve obtained from known concentrations of recombinant LDH from rabbit muscle (Oriental Yeast Co., ltd., Tokyo, Japan). The released ALP activity, an indicator of type II alveolar epithelial cell damage, in the BALF supernatant was measured by the LabAssay™ ALP (FUJIFILM Wako Pure Chemical Corporation, Tokyo, Japan).

### 2.6 Measurement of chemokines and SP‐D in BALF and HO‐1 in lung tissue

The concentrations of cytokine‐induced neutrophil chemoattractant (CINC)‐1 and CINC‐2, a neutrophil chemotactic factor in rats, in the BALF were measured by enzyme‐linked immune‐sorbent assay (ELISA) kits, #RCN100 and #RCN200 (R&D Systems, Minneapolis, MN, USA), respectively. The concentrations of rat SP‐D, a biomarker of lung epithelial damage, in the BALF were measured by an ELISA kit (Yamasa Corporation, Chiba, Japan). All measurements were performed according to the manufacturer's instructions.

The third lobe of the right lung was homogenized with tissue protein extraction reagent (T‐PER) (Thermo Scientific Inc., Rockford, IL, USA) including P8340 (Sigma‐Aldrich, St. Louis, MO, USA), protein inhibitor cocktails, and cOmplete™ Mini (Roche Diagnostics GmbH, Mannheim, Nordrhein‐Westfalen, Germany), protease inhibitor cocktails, and then centrifuged (20 400 g at 4°C for 10 min). The protein concentration of the supernatant was measured by Pierce 660 nm Protein Assay Reagent (Thermo Scientific Inc., Rockford, IL, USA), using bovine serum albumin as a standard. The total protein concentration in the lung tissue was adjusted to a final concentration of 500 mg/ml to measure HO‐1, an index of oxidative stress, which was measured by the ELISA kit ADI‐EKS‐810A (Enzo Life Sciences, Farmingdale, NY, USA).

### 2.7 Histopathology

Formaldehyde‐fixed lung tissue was embedded in paraffin and sectioned at a thickness of 4 μm for hematoxylin and eosin (HE) and Masson trichrome (MT) staining. The slides were assessed for histological changes by a board‐certified pathologist. The Ashcroft scale was used to assess lung fibrosis by score.^4^ We scored the MT‐stained specimens at ×10 magnification with the Ashcroft score, referring to other reports.^5^ The Ashcroft score, briefly explained, is a lung section graded from 0 (normal lung) to 8 (most severe fibrosis), with the grades summed and divided by the number of fields. Immunohistochemical staining for C‐X‐C motif chemokine (CXCL) 5 was performed with rabbit antimouse CXCL5 polyclonal antibody (1:200 dilution, bs‐2549R; Bioss Inc., Woburn, MA, USA), using the lung tissue at 1 month after intratracheal instillation in the control and high‐dose groups. The slides were assessed for histological changes by a board‐certified pathologist.

### 2.8 Statistical analysis

Statistical analysis was carried out using Dunnett's test and Tukey's test, with differences of *P* < .05 considered statistically significant (SPSS, SPSS Inc., Chicago, IL, USA).

## 3 RESULTS

### 3.1 Body and lung weights

**FIGURE 1:**
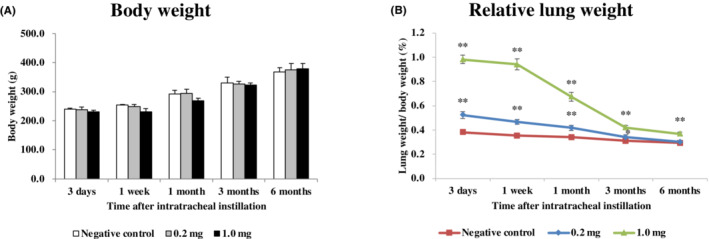
Body weight and relative lung weight (lung weight/body weight). (A) Time course of changes in the body weights of rats in each group. (B) Time course of changes in relative lung weights of rats in each group. Relative lung weight was calculated as a ratio of whole lung weight (g) to body weight (g) for each rat. Each data are presented as mean ± SE (*P < .05, **P < .01). One‐way analysis of variance (ANOVA) followed by Dunnett's test was used to appropriately detect differences between the exposed and control groups.

There were no significant differences in body weight between all the groups (Figure 1A). The relative lung weight (lung weight/body weight) increased in a dose dependent‐manner during the observation period (Figure 1B).

### 3.2 Cell analysis, released LDH activity, and released ALP activity in BALF

**FIGURE 2:**
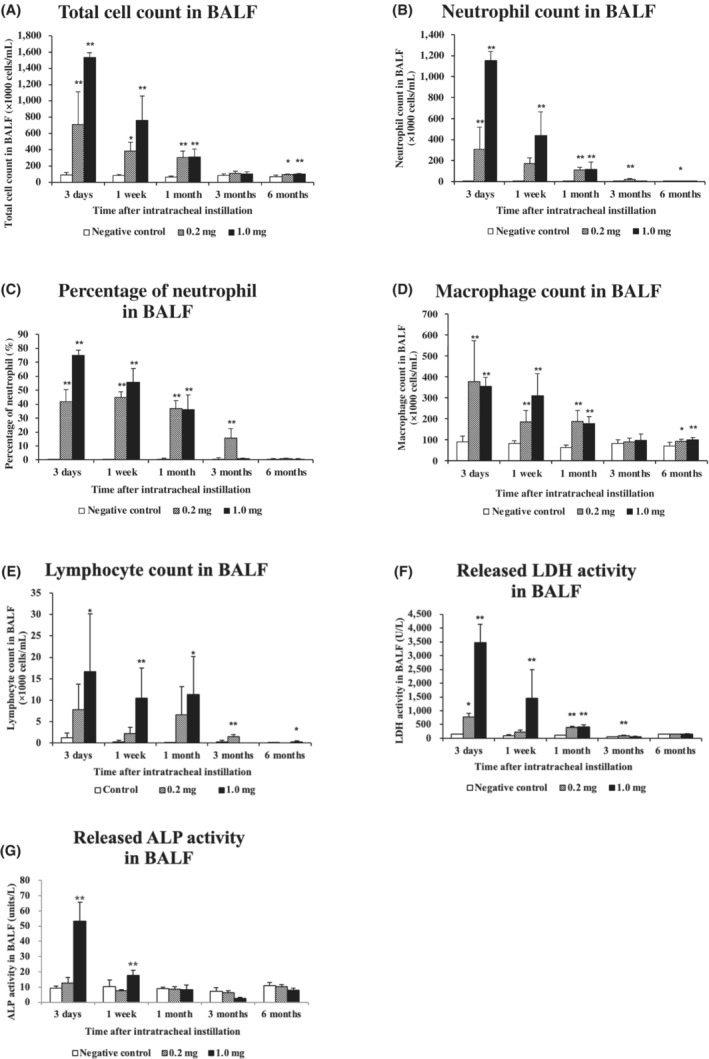
Analysis of cell number, released LDH activity, and released ALP activity in BALF following intratracheal instillation. (A) Total cell count in BALF, (B) neutrophil count in BALF, (C) percentage of neutrophil in BALF, (D) macrophage count in BALF, (E) lymphocyte count in BALF, (F) released LDH activity in BALF, (G) released ALP activity in BALF. Each data are presented as mean ± SE (*P < .05, **P < .01). One‐way ANOVA followed by Dunnett's test was used to appropriately detect differences between the exposed and control groups.

Figure 2 shows the results of inflammatory cell counts, LDH activity, and ALP activity in the BALF. There was a statistically significant increase in the number of total cells in the 0.2 mg and 1.0 mg exposure groups compared with the control group from 3 days to 1 month and at 6 months postexposure (Figure 2A). There were significant increases in the number of neutrophils (Figure 2B) and the percentage of neutrophils (Figure 2C) from 3 days to 3 months postexposure. There was a statistically significant increase in the number of macrophages in the 0.2 mg and 1.0 mg exposure groups compared with the control group from 3 days to 1 month and at 6 months postexposure (Figure 2D). There was a statistically significant increase in the number of lymphocytes from 3 days to 6 months postexposure (Figure 2E). The results of released LDH activity, an index of cell injury, also showed statistically significant increases from 3 days to 3 months postexposure in the exposure groups compared with the control group (Figure 2F). The results of released ALP activity showed statistically significant increases at 3 days and 1 week postexposure in the high‐dose exposure group compared with the control group (Figure 2G).

### 3.3 Concentration of CINC and SP‐D in BALF and concentration of HO‐1 in lung tissue

**FIGURE 3:**
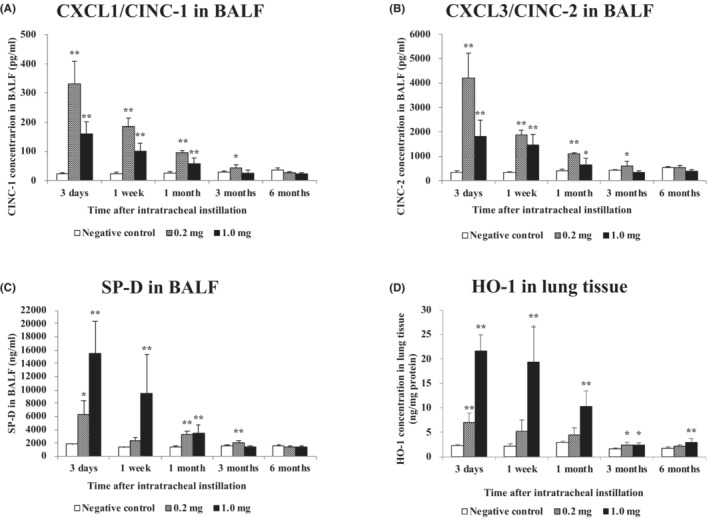
Analysis of chemokines and surfactant protein D (SP‐D) in bronchoalveolar lavage fluid (BALF), and HO‐1 in lung tissue following intratracheal instillation. (A) CXCL1/CINC‐1 in BALF, (B) CXCL3/CINC‐2 in BALF, (C) SP‐D in BALF, and (D) HO‐1 in lung tissue. Each data are presented as mean ± SE (*P < .05, **P < .01). One‐way ANOVA followed by Dunnett's test was used to appropriately detect differences between the exposed and control groups.

Figure 3 shows the concentrations of CINC‐1, CINC‐2, and SP‐D in the BALF and HO‐1 in the lung tissue following the intratracheal instillation of CL‐PAA. The concentration of CINC‐1 and CINC‐2 increased persistently from 3 days until 3 months after exposure (Figure 3A,B). The concentration of SP‐D increased persistently from 3 days until 6 months after exposure (Figure 3C). The concentration of HO‐1 increased persistently from 3 days until 6 months after exposure (Figure 3D). These values tended to decrease with time in the exposed groups.

### 3.4 Histopathological features in the lung

**FIGURE 4:**
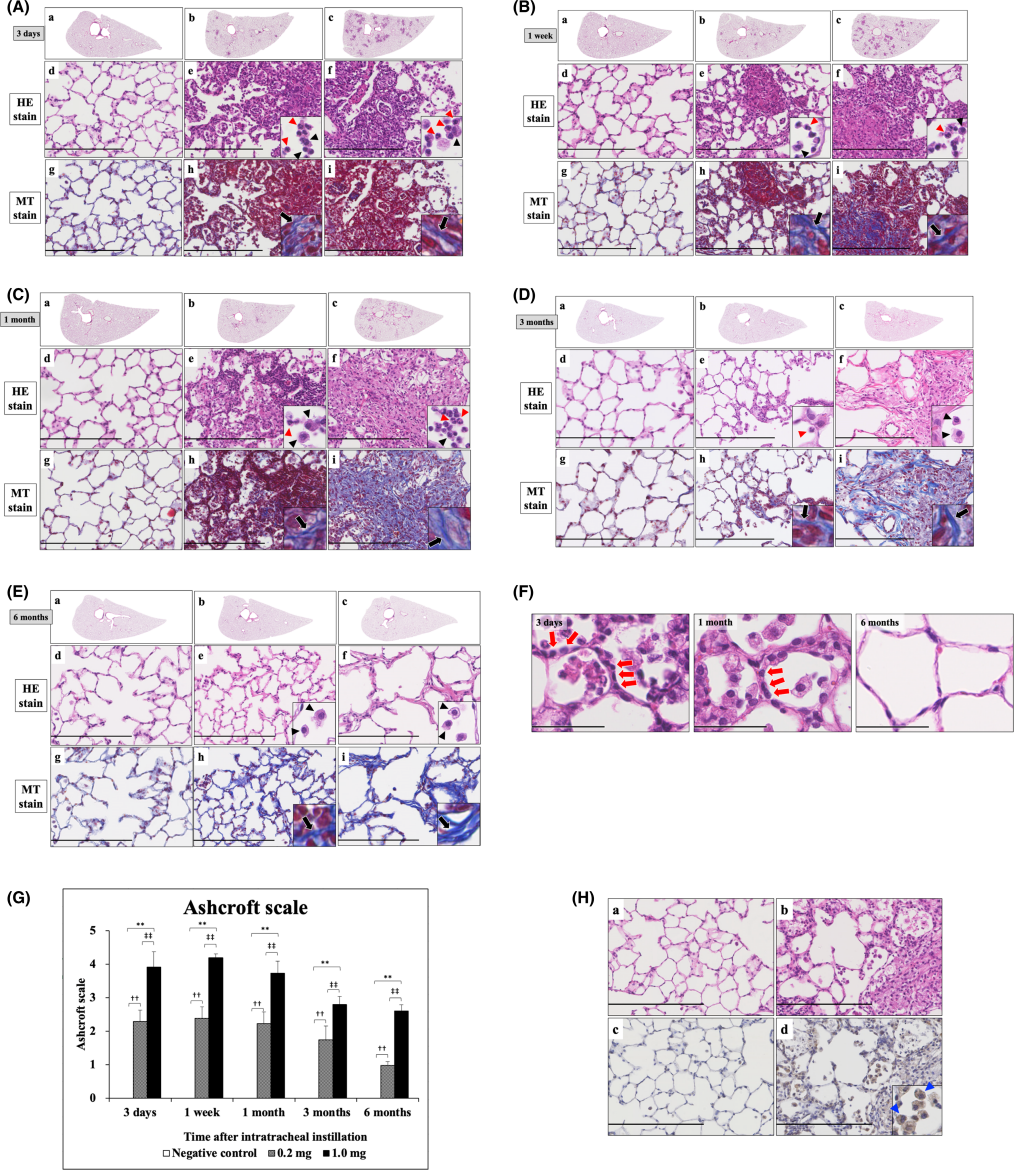
Histological findings following intratracheal instillation (hematoxylin and eosin [HE] and Masson trichrome [MT] staining). Histological findings at (A) 3 days following the instillation (HE and MT staining), (B) 1 week following the instillation (HE and MT staining), (C) 1 month following the instillation (HE and MT staining), (D) 3 months following the instillation (HE and MT staining), (E) 6 months following the instillation (HE and MT staining). (a, d, g) One of the specimens from the group that received distilled water as a control. (b, e, h) One of the specimens from the group that received 0.2 mg of cross‐linked polyacrylic acid (CL‐PAA) as a low dose. (c, f, i) One of the specimens from the group that received 1.0 mg of CL‐PAA as a high dose. Red arrowheads and black arrowheads show neutrophils and macrophages, respectively, in HE staining. Black arrows show collagen fibers in MT staining (internal scale bars in A–E = 250 μm). (F) Histological findings at 3 days, 1 month, and 6 months following the instillation. Red arrows show type II alveolar epithelium‐like cells. There was almost no proliferation of alveolar type II epithelial cells at 6 months (internal scale bars in F, 50 μm). (G) Ashcroft scale at each time. Each data are presented as mean ± SE (*P < .05, **P < .01, ‡*P* < .05, and ‡‡*P* < .01). One‐way ANOVA followed by Tukey's test was used to appropriately detect differences among the exposed groups. (H) Representative images of CXCL5 immunohistochemical staining in lung tissue at 1 month after intratracheal instillation of distilled water as a control group or 1.0 mg CL‐PAA as a high‐dose group. (a) control group (HE staining), (b) high‐dose group (HE staining), (c) control group (CXCL5 immunostaining), (d) high‐dose group (CXCL5 immunostaining). Positive cells of CXCL5 immunohistochemical staining on the high‐dose group were mainly macrophages (blue arrowheads) (internal scale bars in H, 250 μm).

Representative histopathological findings in the lung after intratracheal instillation of CL‐PAA are shown in Figure 4. Inflammatory cell infiltration and edema, mainly neutrophils, were observed in the bronchial wall, type II alveolar epithelium‐like cells increased, fibrin deposition was observed in the alveolar space, and some vitreous membrane formation was also observed, and these changes were dose‐dependent. These are the findings of diffuse alveolar damage (DAD). These changes peaked at 3 days and continued until 3 months later, although they tended to diminish. Fibroblasts and collagen fibrils were also observed in a dose‐dependent manner starting at 3 days, peaking at 1 week, and diminishing but persisting until 6 months later (Figure 4A–F). The Ashcroft scale also showed a significant dose‐dependent increase in the fibrosis score from 3 days, and it persisted until 6 months after exposure (Figure 4G). In the immunohistochemistry of the lungs, CXCL5‐positive cells were found in the high‐dose group 1 month after intratracheal instillation, which was mainly macrophages (Figure 4H).

## 4 DISCUSSION

In this study, we examined that (1) CL‐PAA had continuous inflammatory and fibrosis potentials in the lungs, (2) this inflammation and fibrosis were involved in inflammatory chemokines and oxidative stress, (3) it caused injury to alveolar epithelial cells, and (4) our dosage setting was appropriate.

In the present study, intratracheal instillation of CL‐PAA caused persistent lung inflammation and fibrosis. We had previously conducted intratracheal instillation studies using the same study design to evaluate the inflammatory and fibrotic potential of inorganic materials, in which the results showed that intratracheal instillation of CeO_2_ nanoparticles, which have been reported to cause lung damage such as pulmonary fibrosis and emphysema,^6^, ^7^ caused persistent inflammation in the lungs.^8^ Similarly, persistent inflammation was observed upon intratracheal instillation of NiO nanoparticles with pulmonary toxicity.^9^ On the other hand, in intratracheal instillation studies of TiO_2_ and ZnO, which are considered to be less lung damaging, the inflammation caused by intratracheal instillation was transient.^10^, ^11^ Other reports have similarly found persistent changes in animal models of intratracheal instillation of lung‐damaging inhalants. Ma et al. reported that intratracheal instillation of CeO_2_ into the lungs of rats caused lung inflammation^12^ and fibrosis,^13^ and Luo et al. and Cyphert et al. reported persistent inflammation^14^ and fibrosis^15^ in intratracheal instillation of asbestos, which is known to cause fibrosis in the lungs. Considering that lung damage caused by inorganic materials such as asbestos and crystalline silica can progress from persistent inflammation to irreversible fibrosis and tumors, CL‐PAA, which showed persistent inflammation by intratracheal instillation, was considered to have inflammatory and fibrotic potential in human lungs. Takeda et al. and Suka et al. also performed inhalation exposure and intratracheal instillation of CL‐PAA and reviewed that lung inflammation and fibrosis were observed.^16^, ^17^ Although the physicochemical properties of CL‐PAA are related to the potential for inflammation and fibrosis by CL‐PAA, sufficient physicochemical properties are not shown in their papers.^16^, ^17^ Considering that CL‐PAA induced inflammation and fibrosis in the rat lung in their studies, we speculated that the physicochemical properties of CL‐PAA used in their studies may be partially similar to those in our study.

This study measured CINC‐1 and CINC‐2 concentrations in BALF to examine chemokines involved in persistent inflammation. Intratracheal instillation of CL‐PAA resulted in a sustained and significant increase in both CINC concentrations in the BALF. CINC is a type of CXC chemokine that is induced by macrophages and type II alveolar epithelial cells in response to lipopolysaccharide (LPS) and inflammatory cytokines, such as IL‐1β and TNF‐α,^18^ and it contributes to neutrophil infiltration into the inflammatory site.^19^ We have previously shown that both chemokines are persistently elevated with persistent lung inflammation in CeO_2_ and NiO, which are highly lung‐damaging, while both chemokines are only transiently elevated with transient lung inflammation in TiO_2_ and ZnO, which cause less pulmonary disorder.^20^

The sustained increase in CINC concentration along with the increase in the number of neutrophils in the BALF in this study suggests that CINC may also be involved in the migration of neutrophils in the lungs due to exposure to CL‐PAA. The CINC concentration in this study was independent of the CL‐PAA dose, and the CINC concentration tended to be higher in the low‐dose group. It is possible that the alveoli collapsed due to significant lung inflammation in the high‐dose group and that cytokines in the alveoli were not sufficiently recovered. Moreover, other inflammatory factors, such as oxidative stress, may also be involved in neutrophil migration. In this study, HO‐1 concentration, a marker of oxidative stress,^21^ was also measured in the lung tissue and showed a sustained dose‐dependent increase.

As for other organic materials, Lee et al. reported that PHMG, which caused interstitial pneumonia in Korea, was inhaled by rats and that the expression of 4‐HNE (4‐hydroxynonenal), a marker of oxidative stress, in type II alveolar epithelial cells and other cells increased in a dose‐dependent manner as in lung inflammation, indicating that oxidative stress by organic materials is involved in lung tissue damage, inflammation, and fibrosis.^22^

In this study, fibrosis was observed in the lungs, and CXCL5 may be involved as a mechanism of fibrosis from the results of immunohistochemical staining. We previously reported that elevated expression of CXCL5 may promote epithelial to mesenchymal transition (EMT) and be involved in the onset and persistence of pulmonary fibrosis, and it is possible that those cytokines caused the onset and persistence of lung fibrosis in this experiment.^23^ EMT is a concept regarding the origin of fibroblasts and myoblasts in lung tissue, the idea that lung injury causes epithelial cells to transition to a mesenchymal phenotype, which is thought to involve mainly transforming growth factor beta (TGFβ).^24^

In this study, ALP and SP‐D were measured to examine alveolar epithelial cell injury, and sustained increases in ALP and SP‐D were observed up to 1 month and 3 months, respectively. Since ALP is an enzyme originally present in type II alveolar epithelial cells, its leakage into BALF indicates injury to the type II alveolar epithelium.^25^ The pathological finding in the lungs is DAD, which peaks after 3 days and then declines, consistent with the trend of ALP. On the other hand, SP‐D is a secreted protein produced by type II alveolar epithelial cells and club cells,^26^ so their persistently elevated levels in BALF indicate that type II alveolar epithelial cells are under sustained stimulation.^27^ The HE‐stained images of the pathology in this study showed hyperplasia of type II alveolar epithelium‐like cells peaking at 3 days after intratracheal instillation, and the hyperplasia persisted although DAD reduction was also reduced, suggesting that SP‐D may be produced from these increased alveolar epithelial cells. The difference in persistence of ALP and SP‐D may have been due to the former's release due to injury and the latter's release due to injury and the increase in cells produced by the repair process after that injury.

We set the maximum dose at 1.0 mg/rat to evaluate the material's own pulmonary toxicity in this study. We think that this dose was the maximum dose at which a material with low toxicity induced minimum persistent inflammation in rat lungs following intratracheal instillation. The exposure to 1 mg/rat of fullerene, which is a material with low toxicity and low inflammation, caused a mild sustained neutrophil inflammation, which returned to the level of the negative control in a time‐dependent fashion, in rat lung following intratracheal instillation.^28^ We also previously examined the biopersistence of TiO_2_ nanoparticles with low toxicity among nanomaterials in rat lungs in an intratracheal instillation study, and the clearance of TiO_2_ nanoparticles in the rat lung accompanied by neutrophil inflammation began to delay at doses exceeding 1 mg/rat.^29^ We considered that pulmonary inflammation by a high dose of more than 1.0 mg/rat may be induced by an excessive dose of material, not the toxicity of the material itself. Incidentally, assuming that CL‐PAA would be deposited at the same rate (particle deposition efficiency 0.1, amount of deposited material/1 g of rat lung weight) in rats and humans, the doses of 1.0 mg corresponded to human exposure periods for 1.8 years (calculation in exposure concentration 3 mg/m^3^). As for the particle size, the diameter in the suspension of CL‐PAA is 2.31–3.00 μm, which is less than 4 μm, and it is considered inhalable dust that reaches the lungs' alveoli.

A limitation of this study is that it was an intratracheal instillation study, so the mode of exposure was not physiological. However, since the size of the CL‐PAA in this study was in the category of inhalable dust, the possibility of inducing lung damage such as that seen in this study may well be assumed even when CL‐PAA is exposed by inhalation.

## 5 CONCLUSION

Intratracheal instillation of CL‐PAA‐induced persistent neutrophilic inflammation and fibrosis in the rat lung in a dose‐dependent manner. These results suggest that CL‐PAA has inflammatory and fibrotic potential.

## AUTHOR CONTRIBUTIONS

YH, CN, TT, HI, KY (Kazuhiro Yatera), and YM are responsible for the study design and writing of the manuscript. YH, CN, TT, HI, KW, HH, RO, KS (Kazuki Sumiya), KS (Kazuo Sakurai), KY (Kei Yamasaki), KY (Kazuhiro Yatera), and YM are responsible for data and analysis. CN, TT, HI, HH, RO, KS (Kazuki Sumiya), KS (Kazuo Sakurai), and YM performed the experiments. All the authors read and approved the final manuscript.

## FUNDING INFORMATION

There are no funds that apply.

## DISCLOSURE

Ethical approval: N/A. Informed consent: N/A. Registry and the registration no. of the study/trial: N/A. Animal studies: All procedures and animal handling were done according to the guidelines described in the Japanese Guide for the Care and Use of Laboratory Animals as approved by the Animal Care and Use Committee, University of Occupational and Environmental Health, Japan (animal studied ethics clearance proposal number: AE18‐021). Conflict of interest: We declare no conflict of interest associated with this manuscript.

## Data Availability

The datasets during and/or analyzed during the current study are available from the corresponding author on reasonable request.
